# Synthesis of a Vocal Sound from the 3,000 year old Mummy, Nesyamun ‘True of Voice’

**DOI:** 10.1038/s41598-019-56316-y

**Published:** 2020-01-23

**Authors:** D. M. Howard, J. Schofield, J. Fletcher, K. Baxter, G. R. Iball, S. A. Buckley

**Affiliations:** 1 Department of Electronic Engineering, Royal Holloway, University of London, Egham, Surrey United Kingdom; 2 Department of Archaeology, University of York, The King’s Manor, York, United Kingdom; 3 Leeds Museums and Galleries, Leeds, United Kingdom; 4 Medical Physics Department, Old Medical School, Leeds General Infirmary, Leeds, United Kingdom; 5 Institute for Prehistory, Early History and Medieval Archeology, University of Tübingen, Tübingen, Germany

**Keywords:** Computational models, Electrical and electronic engineering

## Abstract

The sound of a 3,000 year old mummified individual has been accurately reproduced as a vowel-like sound based on measurements of the precise dimensions of his extant vocal tract following Computed Tomography (CT) scanning, enabling the creation of a 3-D printed vocal tract. By using the Vocal Tract Organ, which provides a user-controllable artificial larynx sound source, a vowel sound is synthesised which compares favourably with vowels of modern individuals.

## Introduction

The sound of a vocal tract from the past has been synthesised to be heard again in the present, allowing people to engage with the past in completely new and innovative ways. The precise dimensions of an individual’s vocal tract produce a sound unique to them^1^. If the tract dimensions can be scientifically established, vocal sounds can be synthesised by using an electronic larynx sound source^2^ and a 3-D printed vocal tract^3^. Since the restoration of an exact vocal sound requires the perfect preservation of the soft tissues, this is impossible for individuals whose remains are only skeletal. Even where soft tissue does survive, for example in mummified remains^4^, the vocal tract can either be missing or distorted^5^. The process is only feasible when the relevant soft tissue is reasonably intact, as in the case of the 3,000 year-old mummified body of the Egyptian priest Nesyamun^6^, whose ‘in death’ vocal tract acoustic output has been scientifically synthesised. This acoustic output is for the single sound for the extant vocal tract shape; it does not provide a basis for synthesising running speech. To do so would require knowledge of the relevant vocal tract articulations, phonetics and timing patterns of his language. The synthesised vowel sound based on the precise dimensions of his unique vocal tract is here compared to modern vowels as proof of method and to demonstrate future research potential.

Having established the scientific recreation of a 3-D printed vocal tract unique to a living individual, the ‘Voices from the Past’ Project was set up to investigate this possibility for those long dead in cases where their remains are sufficiently well preserved. With the need for optimum preservation of the vocal tract an essential requirement, combined with the practical necessity for precise CT-imaging in close proximity to the individual selected, the mummified body of Nesyamun was a highly appropriate choice. This was also true for archaeological reasons.

**Figure 1:**
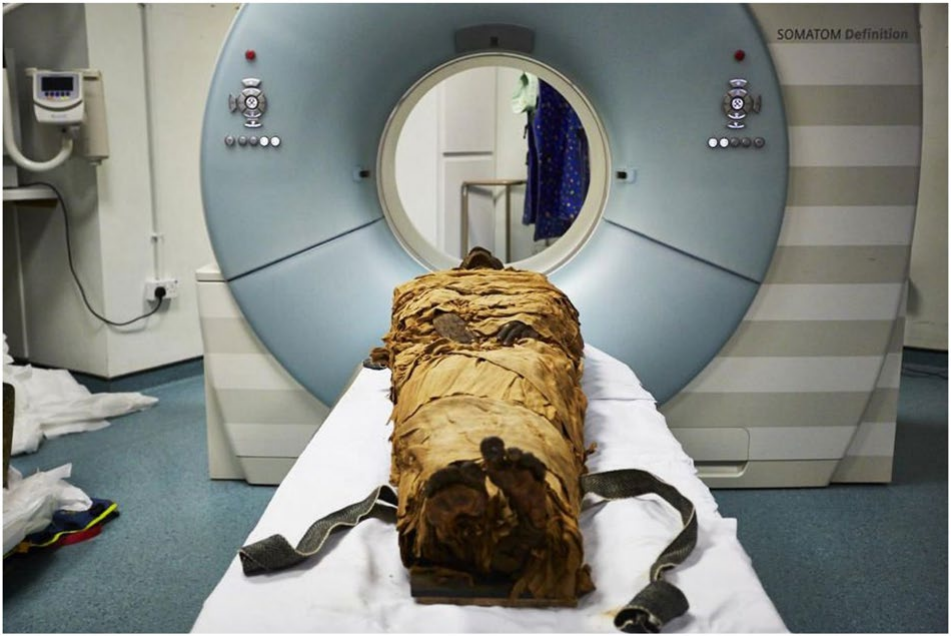
The mummified body of Nesyamun laid on the couch to be CT scanned at Leeds General Infirmary. © Leeds Teaching Hospitals/Leeds Museums and Galleries.

The Egyptian Nesyamun (Fig. 1) lived during the politically volatile reign of pharaoh Ramses XI (c.1099–1069 BC) over 3000 years ago, working as a scribe and priest at the state temple of Karnak in Thebes (modern Luxor). His voice was an essential part of his ritual duties which involved spoken as well as sung elements^7^.

With his mummified remains now displayed in Leeds City Museum, the current project is only the most recent to examine Nesyamun, whose remains have been at the forefront of mummy studies for almost two centuries. Following the unwrapping of his body in 1824, it was examined by members of the Leeds Philosophical and Literary Society including three surgeons and a chemist whose multidisciplinary scientific investigation published in 1828^8^ was the first of its kind. Following the development of X-rays, the body underwent radiological examination in 1931 at the University of Leeds’ School of Medicine, in 1964 by the University of Sheffield School of Dentistry, and in 1990 at the University of Manchester by a team using endoscopy, histology, X-ray and early CT scanning techniques^9,10^. These combined studies revealed that Nesyamun had died in his mid-50s^9^ and had suffered from gum disease and severely worn teeth, yet nonetheless “had a strong well-developed mandible”, which, like the maxilla, was ‘prognathic’, and “clearly Nubian blood had once coursed through his veins”^11^.

**Figure 2:**
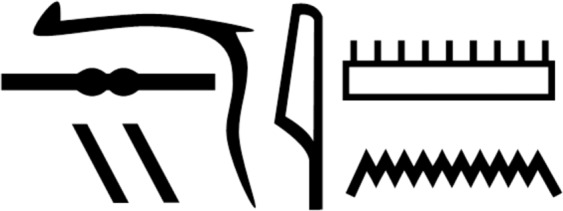
Nesyamun’s name in hieroglyphs as shown in his coffin inscriptions.

His coffin inscriptions give the name Nesyamun (Fig. 2)^7^, but as one of the first ancient Egyptian names to be translated following the decipherment of hieroglyphs in 1822, this was initially read ‘Natsif-Amon’^8^ with at least nine later variants^9,12^ until eventually corrected to Nesyamun^9^. This was a vital clarification within ancient Egyptian culture in which the name was regarded as essential to an individual as their physical (mummified) body and their soul (ka) and spirit (ba). It was also a fundamental belief that ‘to speak the name of the dead is to make them live again’ (alternatively translated: ‘a man is revived when his name is pronounced’^13^), both by living relatives and by the deceased themselves when appearing before the gods of judgement. Only those able to verbally confirm that they had led a virtuous life were granted entry into eternity and awarded the epithet ‘maat kheru’, ‘true of voice’^14^, as applied to Nesyamun himself throughout his coffin inscriptions. In these texts, Nesyamun asks that his soul receives eternal sustenance, is able to move around freely and to see and address the gods^9^ as he had in his working life. Therefore his documented wish to be able to speak after his death, combined with the excellent state of his mummified body, made Nesyamun the ideal subject for the ‘Voices from the Past’ project for which his body was re-examined using state-of the-art CT scanning equipment.

Since human remains have unique status not as ‘objects’ but as the remains of once-living people (see SI), it was also necessary to consider the ethical issues raised by the research and its possible heritage outcomes^15,16^. The team concluded that the potential benefits outweighed the concerns, particularly because Nesyamun’s own words express his desire to ‘speak again’ and that the scientific techniques used were non-destructive.

**Figure 3:**
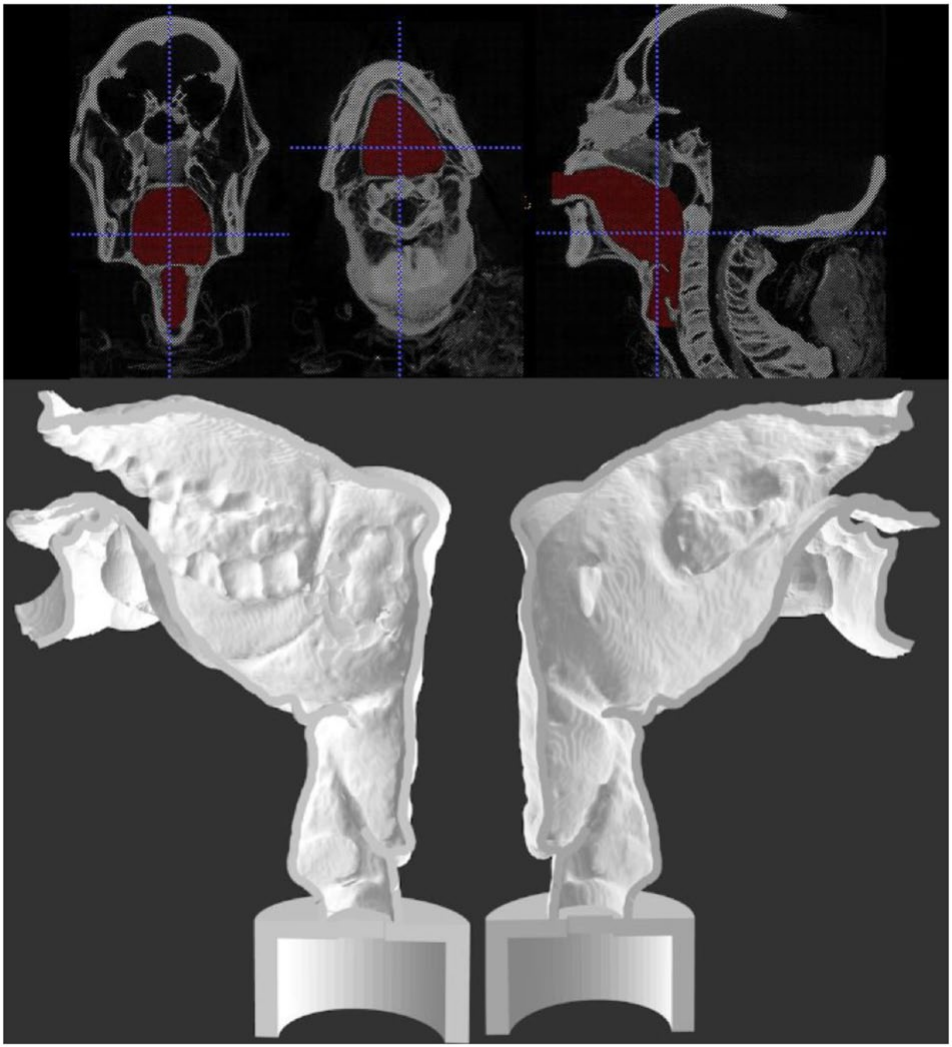
Final segmentation view (upper) and sagittal section of the two halves of 3-D printed Nesyamun’s vocal tract (lower). The lack of tongue muscular bulk and soft palate is clear.

The CT images confirmed that a significant part of the structure of Nesyamun’s larynx and throat remains *in situ* as a result of the elaborate mummification process, thus enabling the vocal tract shape to be measured. The tongue, however, has lost its muscular bulk over time and the soft palate is not present as illustrated in Fig. 3. The dimensions of Nesyamun’s tract are 81.4 mm between the external front of the upper lip and the hard/soft palate boundary and 68.4 mm between the thyroid notch and the hard/soft palate boundary. Comparable measurements for two living adult males are 103.6/111.0 mm and 80.0/86.0 mm respectively. Nesyamun’s tract therefore appears notably smaller than those of contemporary adult males.

Following the scans, a 3-D printed tract was created for Nesyamun and designed to be used with the Vocal Tract Organ^17^ which provides an appropriate acoustic larynx source as a time domain waveform synthesis of the Liljencrants-Fant (LF) larynx source which is commonly employed in speech synthesis^18^. The fundamental frequency, loudness and vibrato rate and depth can be individually controlled. The tract incorporates a coupler at its larynx end that is designed to fit snugly over the output end of an Adastra model 952-210 (16 ohm, 60 Watt) loudspeaker drive unit.

**Figure 4:**
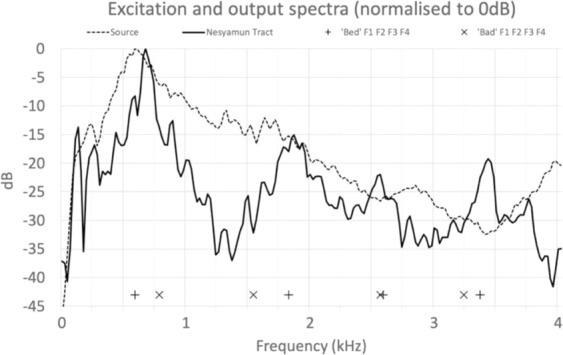
Long-term average spectra for (**a**) the Vocal Tract Organ output larynx source (dashed), and (**b**) the output from the 3-D printed vocal tract for Nesyamun acoustically excited by the Vocal Tract Organ output (solid). The first four formant positions averaged from six male English speakers are shown for the words ‘bad’ (+) and ‘bed (x).

Figure 4 shows long-term average spectra for (1) the source signal from the Vocal Tract Organ via the Adastra loudspeaker alone (dotted line) and (2) the output from the 3-D printed vocal tract for Nesyamun placed atop the Adastra loudspeaker (solid line). The joystick controlled version of the Vocal Tract Organ was used to create a larynx input to the 3-D printed vocal tract of Nesyamun and the joysticks were not altered during the sound. Four formant peaks are evident in the output spectrum for Nesyamun’s 3-D printed vocal tract which are indicative of four resonances in Nesyamun’s vocal tract within this frequency range.

**Figure 5:**
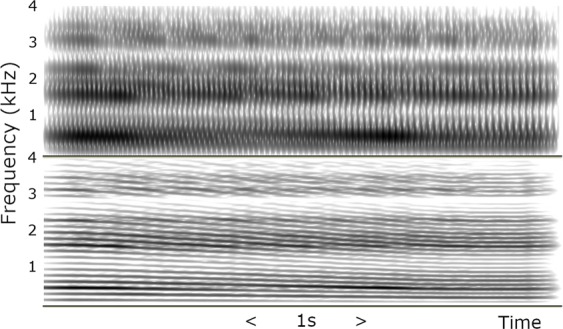
Wide band 30 ms window (upper) and narrow band 5 ms window (lower) spectrograms for a quasi-spoken falling intonation generated using a joystick controlled Vocal Tract Organ driving the 3-D printed vocal tract for Nesyamun.

Of vital perceptual importance in recreating a natural vocal sound is the application of some form of fundamental frequency variation. Nesyamun’s duties included speaking as well as chanting or singing the daily liturgy^7^, so the vocal tract organ was used to provide a falling intonation in the male speech fundamental frequency range. Narrowband (30 ms window) and wide-band (5 ms window) spectrograms for a few vibrato cycles of the output are shown in Fig. 5 and formant frequency values averaged throughout the sound measured using Praat^19,20^ are shown in Table 1.

**Table 1:** Measured average frequencies for formants one to four (F1–F4) throughout the sound created with the 3-D printed vocal tract for Nesyamun excited by the Vocal Tract Organ.

	F1 (Hz)	F2 (Hz)	F3 (Hz)	F4 (Hz)
Nesyamun tract	690	1870	2560	3420
Mean for 6 males ‘bed’	594	1821	2578	3350
Mean for 6 males ‘bad’	783	1542	2559	3227

Comparison data of averaged first four formant frequencies for six adult male English speakers for the vowels in ‘bed’ and ‘bad’ for comparison.

The measured lower three formant values for the vowel of Nesyamun fall between the vowel in ‘bed’ and the vowel in ‘bad’ within the formant data quoted in the classic 1952 work by Peterson and Barney^21^, based on the closeness in frequency of the second and first formants respectively. By way of a modern comparison, the first four formant frequencies averaged for six adult male English speakers for the vowels in ‘bed’ and ‘bad’, measured using Praat, are given in Table 1 and plotted in Fig. 4. The match for F1 and F3 is remarkably good but there are differences for F2 and F4. There will not be an exact match because: (1) no two vocal tracts are exactly the same so there will always be formant frequency differences between speakers, (2) any acoustic similarity between a modern English pronunciation of ‘bed’ and bad’ and the language of Nesyamun cannot be assumed, and (3) Nesyamun’s vocal tract posture is not set for speaking any specific vowel; rather it is set appropriate for his burial position. In addition, his tongue has lost much of its muscle bulk and his soft palate is missing.

Aside from the archaeological possibilities based on the relatively rare survival of intact vocal tract soft tissue as in the case of Nesyamun, it is possible to estimate vocal tract shapes purely from skeletal information^22^ via, for example, ellipses of dispersion for skull bony landmarks^23^. Making use of 3-D printed vocal tracts is one way to recreate the sound output; it is also feasible to calculate the acoustic output based on digital waveguides^24^ and this offers a future possibility of producing a dynamic acoustic output of spoken or sung utterances.

Previous attempts to recreate the voice of an ancient individual have employed software techniques to animate a facial reconstruction image to give an approximation of the original voice^25,26^. Recordings do exist of individuals with extraordinary voices who died soon after the introduction of sound recording, such as the last castrato (Alessandro Moreschi - recorded in 1902 and 1904)^27^, but here is offered a vocal recreation that is based on an extant vocal tract preserved over three millennia. This innovation has implications for the way in which the past is presented to the public, either through conventional heritage interpretation displays or via digital interventions.

As a rare witness to a cataclysmic period in Egypt’s ancient history, Nesyamun also has a pre-eminent place in the history of Egyptology. His body and coffin have been on permanent display in Leeds Museum for almost two centuries, and although few visitors can read his coffin’s hieroglyphic texts for themselves, the possibility of transmitting their vocalisation would not only fulfil Nesyamun’s own wishes as he himself expressed, but make them accessible to all^28^. Having considered and accommodated all ethical implications, the transmission of sound resulting from his actual vocal tract after a three millennia silence would mean that those who come to see him would also be able to *hear* a sound from his vocal tract as an initial step, emphasising his humanity with the potential to excite and inspire.

Similarly, the well-preserved temple of Karnak in which Nesyamun undertook his duties is the destination for over a million visitors each year, providing further exciting possibilities for heritage interpretation within Egypt’s tourist economy.

This innovative interdisciplinary collaboration has produced the unique opportunity to hear the vocal tract output of someone long dead by virtue of their soft tissue preservation and new developments in technology, digital scanning and 3-D printing. While this approach has wide implications for heritage management/museum display, its relevance conforms exactly to the ancient Egyptians’ fundamental belief that ‘to speak the name of the dead is to make them live again’. Given Nesyamun’s stated desire to have his voice heard in the afterlife in order to live forever, the fulfilment of his beliefs through the synthesis of his vocal function allows us to make direct contact with ancient Egypt by listening to a sound from a vocal tract that has not been heard for over 3000 years, preserved through mummification and now restored through this new technique.

## Methods

In September 2016 Nesyamun’s mummified body was transferred from Leeds City Museum to the nearby Computed Tomography (CT) Scanning Department at Leeds General Infirmary. Once within the scanning room it was removed from its coffin and transferred onto the couch of a Siemens Definition (Erlangen, Germany) multi-detector CT scanner. Positioned on the couch in a head-first supine orientation (Fig. 1), a high resolution helical CT scan was performed from which contiguous axial images of 0.6 mm slice thickness were reconstructed covering the range from cranial vertex to hallux. Images were acquired at a tube voltage of 120kVp, tube current-time product of 180mAs, detector coverage of 64 × 0.6 mm and helical pitch factor of 1.0 and were reconstructed using a soft tissue convolution kernel and a 450 mm reconstructed field of view. In order to improve visualisation of the vocal tract, a further set of axial, coronal and sagittal images covering cranial vertex to lung apices were reconstructed using a 0.6 mm slice thickness and 220 mm reconstructed field of view. All image sets were exported in uncompressed DICOM format for further manipulation and processing.

ITK-SNAP^29^, which allows a three-dimensional structural representation of human tissues to be observed, was used to view the airway between the larynx and lips which is itself isolated as a solid shape to enable the 3-D printing process. On-screen, air is generally represented in black, with volumes of soft tissue and bone being represented in grey to white. The process of creating the vocal tract model itself involves semi-automatic growing’ of user-defined starting spheres within the black (air) volumes outwards to stop at soft tissue/bone boundaries which are denoted by a change in contrast. This process involves trial and error alongside close observation of the determined boundaries. Making changes to the starting positions of the user-defined spheres and repeating the process as necessary is a core element of this process. Post-processing hand-editing of the final airway with direct reference to the original CT data enables final minor changes to be made as appropriate. In particular, the lack of a soft palate (see Fig. 3) meant that its position had to be estimated prior to printing.

The resulting airway volume represents the inside of Nesyamun’s vocal tract as it is preserved. Whilst the vocal tract soft tissue is essentially intact and the oral and pharyngeal cavities are well represented (see Fig. 3), the tongue is desiccated therefore losing the majority of its bulk. In addition, the soft palate is missing and the tract boundary it normally forms has therefore been estimated. A virtual sheath is created around the airway to which a loudspeaker coupler is added. The resulting vocal tract model is 3-D printed (Stratysys Connex 260 machine – 200 micron maximum linear printing error at this scale).

## Supplementary information

Supplementary information 

Supplementary video

## Data Availability

The data supporting the findings are fully available without restriction. Relevant data are available from the corresponding author upon request.
