# Supplementary material for: Synthesis of a Vocal Sound from the 3,000 year old Mummy, Nesyamun ‘True of Voice’
Source: Sci Rep. 2020 Jan 23;10:45000. doi: 10.1038/s41598-019-56316-y (PMC6978302; doi:10.1038/s41598-019-56316-y)
Supplement: Supplementary file 1 — Supplementary information [file 41598_2019_56316_MOESM1_ESM.pdf]

Synthesis of a Vocal Sound from the 3,000 year old Mummy, Nesyamun 'True of Voice'

Howard, D.M.[1,] Schofield, J.[2], Fletcher, J.[2], Baxter, K.[3], Iball, G.[4], and Buckley, S.A.[2,5]

[1]Department of Electronic Engineering, Royal Holloway, University of London, Egham, Surrey, United Kingdom
[2]Department of Archaeology, University of York, The King's Manor, York, United Kingdom
[3]Leeds Museums and Galleries, Leeds, United Kingdom
[4]Medical Physics Department, Old Medical School, Leeds General Infirmary, Leeds, United Kingdom
[5]Institute for Prehistory, Early History and Medieval Archaeology, University of Tübingen, Tübingen, Germany

*john.schofield@york.ac.uk, David.Howard@rhul.ac.uk.

Supplementary Information

Additional historical context

The inscriptions on Nesyamun's coffin (Fig. S1) were finally translated and published in 2008 by the Leeds Philosophical and Literary Society. They note that "it is most gratifying to see the collections of the Old Philosophical Hall on display again ... especially the Leeds Mummy with its 3,000 year old message to the gods of antiquity" (Hatton in Wassell 2008, preface). Nesyamun's coffin inscriptions are predominantly made up of prayers from the Book of the Dead funerary texts.

The project also provided a unique opportunity to further study one of the most iconic attractions in Leeds City Museum, and add to current understanding through a full-body CT scan.

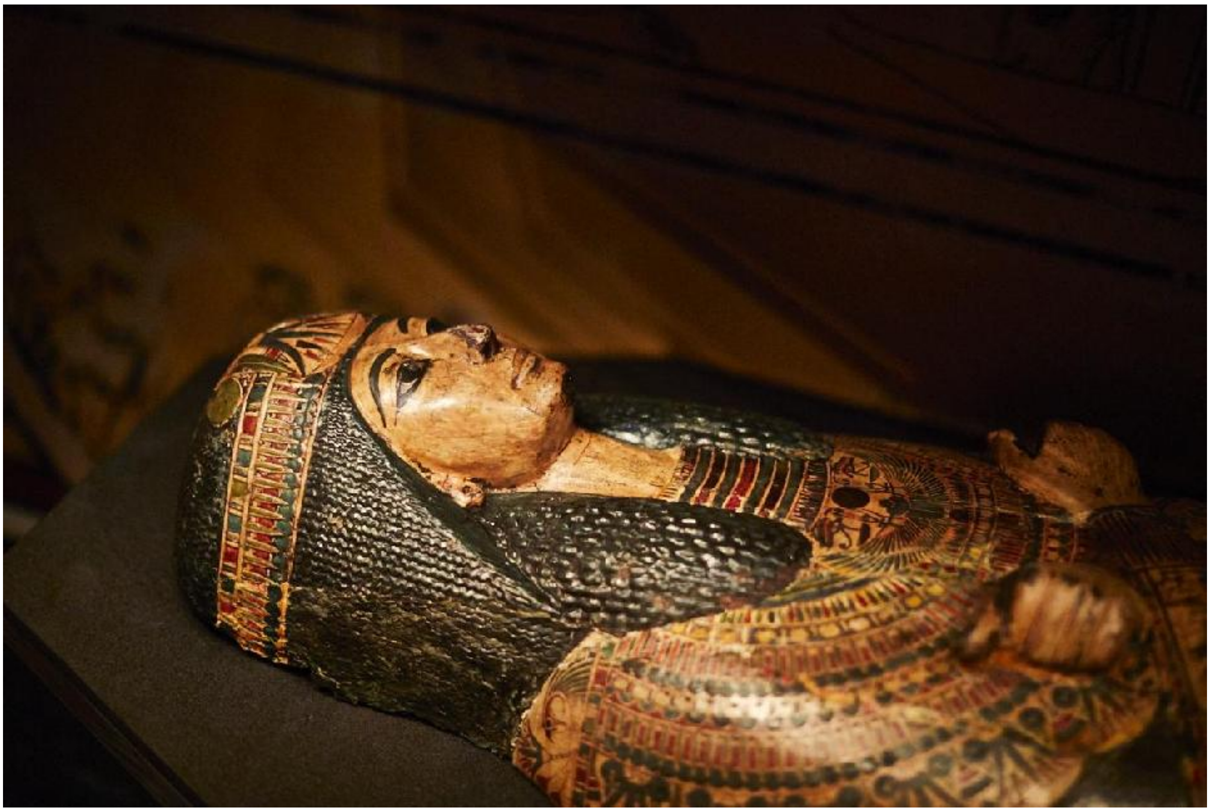

Figure S1: Upper part of the inner coffin lid of Nesyamun. © Leeds Museums and Galleries.

Ethics

The team (a unique collaboration involving an electronic engineer, Egyptologist, archaeologist, museum curator, archaeological scientist and clinical scientist) considered the ethical issues that such research might raise plus the ethical implications of any potential heritage outcomes. Since Leeds Museums and Galleries (LMG) are custodians of the city's human remains collection, they are responsible for ensuring Nesyamun's preservation and for generating research and interpretation for the public in accordance with ethical best practice. Acknowledging that human remains have unique status within museum collections, not as 'objects' but as the remains of once-living people, their Human Remains Policy states that "LMG will provide access to and use human remains and images of human remains in a respectful and culturally appropriate, sensitive and informative manner". So in accordance with the DCMS Guidance for the Care of Human Remains in Museums (2005), LMG's Human Remains Working Group discussed the risks, benefits and ethics of the current scientific study of Nesyamun at all stages of the project, concluding that the potential benefits outweighed the concerns, particularly because the scientific techniques used were non-destructive. The project provided a unique opportunity to further study one of the most iconic attractions in Leeds City Museum, and add to current understanding through a full-body CT scan. The potential future heritage outcomes for the public will also be subject to ethical scrutiny.

It is recognised, however, that using a synthesised voice from a deceased person has its own ethical implications, and these would need to be discussed by Leeds Museums and Galleries in accordance with their Human Remains Policy.

Modern applications

A further possibility for the future lies in giving back vocal sounds to those who lose normal vocal fold function of their larynx (e.g. following surgery for laryngeal cancer) or vocal tract function (e.g. following physical trauma) which would render natural pitch and/or vocal tract shape articulation unusable respectively. Those suffering the former resort to alaryngeal voice production via their post-laryngectomy vocal tract such as members of the Alaryngeal Choir (www.shoutatcancer.org). Where pre-laryngectomy vocal tract MRI images are available the possibility exists to apply Vocal Tract Organ larynx excitation to enable a vocal hark-back choral sound from alaryngeal choristers.

Creating 3-D printed vocal tracts is based on a technique proposed by Bertrand Delvaux. The tract for Nesyamun (Fig S2) and an increasing assortment of tracts will provide the Vocal Tract Organ with its stops to enable musical performances.

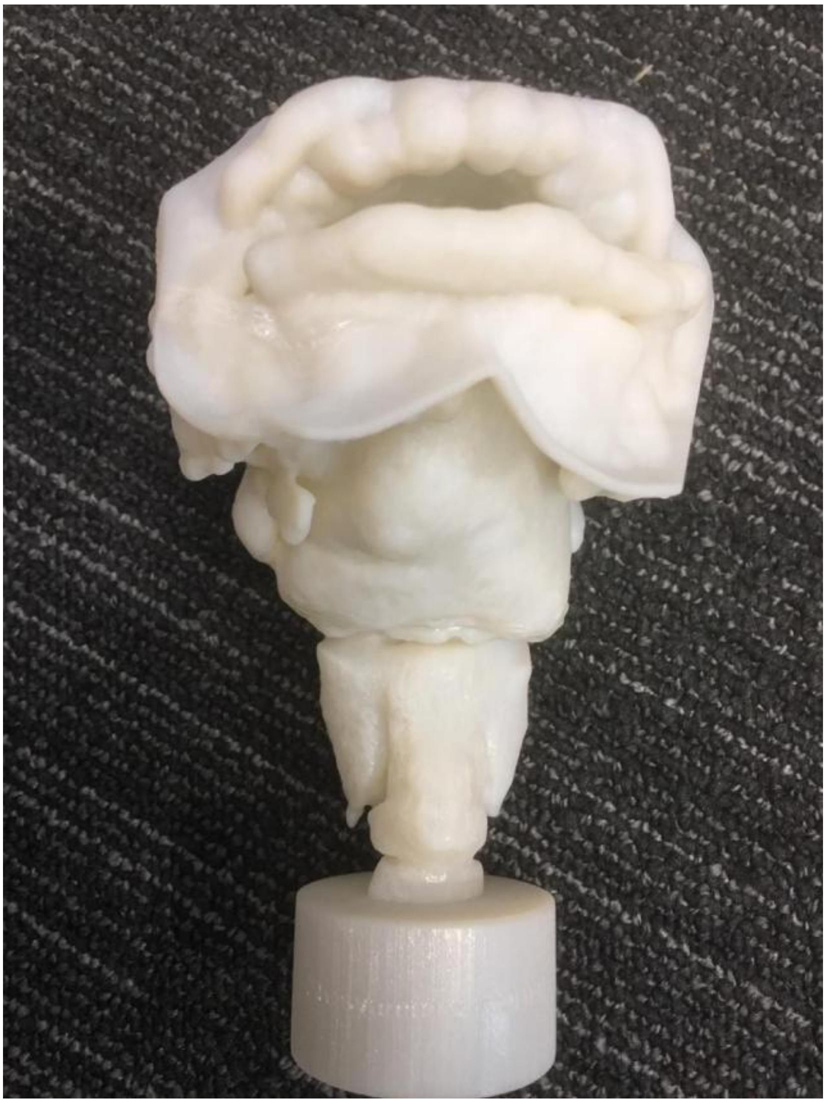

Figure S2: 3-D printed vocal tract of Nesyamun
